# Regulation of cellular senescence *via* the FOXO4‐p53 axis

**DOI:** 10.1002/1873-3468.13057

**Published:** 2018-05-13

**Authors:** Benjamin Bourgeois, Tobias Madl

**Affiliations:** ^1^ Gottfried Schatz Research Center for Cell Signaling, Metabolism and Aging, Molecular Biology and Biochemistry Medical University of Graz Austria; ^2^ BioTechMed Graz Austria

**Keywords:** *FOXO*, *p53*, *senescence*

## Abstract

Forkhead box O (FOXO) and p53 proteins are transcription factors that regulate diverse signalling pathways to control cell cycle, apoptosis and metabolism. In the last decade both FOXO and p53 have been identified as key players in aging, and their misregulation is linked to numerous diseases including cancers. However, many of the underlying molecular mechanisms remain mysterious, including regulation of ageing by FOXOs and p53. Several activities appear to be shared between FOXOs and p53, including their central role in the regulation of cellular senescence. In this review, we will focus on the recent advances on the link between FOXOs and p53, with a particular focus on the FOXO4‐p53 axis and the role of FOXO4/p53 in cellular senescence. Moreover, we discuss potential strategies for targeting the FOXO4‐p53 interaction to modulate cellular senescence as a drug target in treatment of aging‐related diseases and morbidity.

## Abbreviations

**ASPP2**, apoptosis‐stimulating of p53 protein 2

**ATM**, ataxia‐telangiectaxia mutated kinase

**Bcl‐xL**, B‐cell lymphoma‐extra large

**BRAF**, B‐raf proto‐oncogene

**CBP**, CREB‐binding protein

**Cdk**, cyclin‐dependent kinase

**CDKi**, cyclin‐dependent kinase inhibitor

**Chk1**, downstream checkpoint kinases 1

**Chk2**, downstream checkpoint kinases 2

**CK1**, casein 1‐like kinase

**CMEC**, cerebral microvascular endothelial cells

**DBD**, DNA‐binding domain

**DBE**, daf‐16 family member‐binding element

**DDR**, DNA‐damage response

**DRI**, D‐retro‐inverso

**ERK1**, extracellular signal‐regulated kinases 1

**ERK2**, extracellular signal‐regulated kinases 2

**FH**, forkhead domain

**FOXO**, forkhead box O

**HAT**, histones acetyl‐transferase

**HIPK2**, homeodomain‐interacting protein kinase 2

**HKa**, cleaved high molecular weight kinogen

**IR**, ionizing‐radiation

**IRE**, insulin‐responsive sequence

**JNK**, c‐jun N‐terminal kinase

**MAPK**, mitogen‐activated protein kinase

**MDM2**, mouse double mutant 2 homolog

**MEF**, mouse embryonic fibroblast

**MEK**, mitogen‐activated protein kinase kinase

**MnSOD**, Manganese superoxide dismutase

**NMR**, nuclear magnetic resonance

**NRD**, negative regulatory domain

**OIS**, oncogene‐induced senescence

**PI3K**, phosphatidylinositol 4,5‐biphosphate 3‐kinase

**PML**, promyelocytic leukemia

**PRAK**, p38‐regulated/activated protein kinase

**PTEN**, phosphatase and tensin homolog

**PTM**, post‐translational modification

**Rb**, retinoblastoma protein

**RNAPII**, RNA polymerase II

**ROS**, Reactive‐oxygen species

**SGK1**, serum/glucocorticoid regulated kinase 1

**SIRT1**, sirtuin‐1

**TAD**, trans‐activation domain

**TD**, tetramerization domain

**USP7**, ubiquitin specific peptidase 7

Cellular senescence refers to a permanent cell‐cycle arrest during which cells are unable to re‐enter the cell‐cycle despite the presence of growth‐factors therefore limiting the lifespan of mammalian cells and preventing unlimited proliferation. Cellular senescence is beneficial during normal embryonic development and upon tissue damage by promoting tissue remodelling and renewal (for review 1). Moreover, cellular senescence has been shown to play important roles in wound healing as well as cardiac and liver fibrosis 2, 3, 4. Senescence can be triggered by various cellular stresses including telomere loss, oxidative stress or intense oncogenic signalling 5, 6, 7. It was first associated with a declining frequency of cell replication over age and therefore named replicative senescence 8, 9. Replicative senescence is a result of telomere shortening during cell proliferation that is recognized by the cell as double‐strand break. This triggers DNA‐damage response (DDR) leading to Ataxia‐telangiectaxia mutated kinase (ATM) mediated activation of p53 and cell‐cycle arrest 10, 11, 12. Intense oncogenic signalling has also been shown to trigger cellular senescence, resulting in decreased tumour development and therefore considered as a defensive mechanism against cancer 13. This process is known as oncogene‐induced senescence (OIS). Persistent activation of oncogenic processes during early tumorigenesis triggers cell hyper‐proliferation resulting in enforced DNA replication and accumulation of reactive‐oxygen species (ROS) 14. Similarly than for the replicative senescence the DDR is then activated and leads to cellular senescence *via* the cooperation of several tumour suppressor networks. Three cyclin‐dependent kinase inhibitors (CDKi), p16, p21 and p27 have been shown to accumulate in senescent cells and drive cell‐cycle arrest and subsequent cellular senescence (for review 15, 16). Mechanistically, p16 and p21 maintain the retinoblastoma protein (Rb) in its active hypo‐phosphorylated form which in turn activates the E2F family of transcription factors. The latter mediate the transactivation of genes coding for key components of the cell cycle machinery. Importantly, the expression of these CDKi differs at different stages of cellular senescence. While p16 is maintained at high expression level, p21 appears to accumulate only at early stages suggesting different functions in either induction or maintenance of cellular senescence 17. Despite the lack of molecular evidences on the exact pathways involved in cellular senescence, p53 and Forkhead box O (FOXO) proteins seem to play a central role, especially in triggering the accumulation of p21 in senescent cells.

This review will aim to describe both the detailed molecular mechanisms by which FOXO and p53 on their own, and jointly, regulate cellular senescence. A particular emphasis will be on the structural work carried out with the purified proteins to decipher their complex interactions. Moreover, we will also discuss the potential role of targeting the FOXO‐p53 regulatory axis in order to develop therapeutic approaches for treating aging‐related diseases and morbidity.

## p53 and cellular senescence

**Figure 1:**
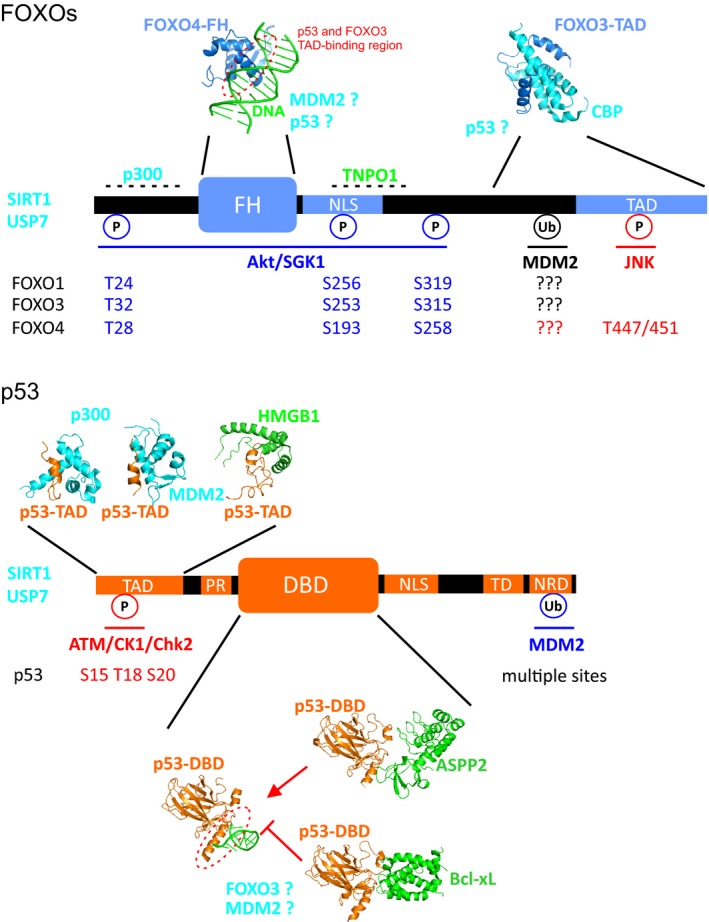
Structural organization of FOXOs and p53 and post‐translation modifications involved in senescence. Forkhead box class “O” (FOXO) proteins are composed of an N‐terminal Forkhead domain (FH) followed by a nuclear localization signal (NLS) and a C‐terminal transactivation domain (TAD). p53 is composed of an N‐terminal TAD domain, a proline‐rich domain (PR) followed by a DNA‐binding domain (DBD), a NLS, a C‐terminal tetramerization domain (TD) and a negative regulatory domain (NRD). Most relevant post‐translationally modified residues in p53 and FOXOs and the corresponding enzymes involved are annotated in blue, red or black if they are either involved in inactivation, activation or both, respectively. FOXO and p53 binding partners discussed in this review are indicated in cyan or green, depending on whether they are common or specific to FOXOs and/or p53. The available three dimensional structure of the corresponding complexes are shown in cartoon (FOXO4/DNA: PDB ID 3L2C 57; FOXO3/CBP: 2LQH 64; p53/DNA: 3IGK 125; p53/MDM2: 1YCR 35; p53/HMGB1: 2LY4 22; p53/p300: 2MZD 107; p53/ASPP2: 1YCS 29; p53/Bcl‐xL: 2MEJ 30).

p53 is a homo‐tetrameric transcription factor composed of an N‐terminal trans‐activation domain (TAD, residues 1–61), a prolin‐rich domain (PR, residues 64–92), a central DNA‐binding domain (DBD, residues 93–293) followed by a tetramerization domain (TD, residues 325–355) and the C‐terminal negative regulatory domain (NRD, residues 367–393) (Fig. 1). p53 is involved in the regulation of more than 500 target genes and thereby controls a broad range of cellular processes, including metabolic adaptation, DNA repair, cell cycle arrest, apoptosis, and senescence (for review 18). The multifaceted function of p53 is closely related to its multi‐functional domain architecture making p53 a scaffold molecule acting as a central protein‐protein interaction hub in a plethora of biological networks. These p53 interaction partners allow for tight regulation of p53 localization, stability and transcriptional activity. Biochemical and structural biology work revealed that these interaction partners share common binding motifs on p53 and through this modulate p53 function and activity (for review 19). The N‐terminal p53 transactivation domain, for example, is largely disordered but possesses two binding motifs with α‐helical propensity, named TAD1 (residues 17–29) and TAD2 (residues 40–57). These two motifs act independently or in combination in order to allow p53 binding to several proteins regulating either p53 stability, such as trough the mouse double mutant 2 homolog (MDM2) 20, or transcriptional activity, such as trough binding of the transcriptional coactivator p300 21 or high mobility group B1 (HMGB1) 22. As an additional layer of regulation of p53 activity the TAD domain is highly phosphorylated in cells by several kinases including ATM 23, casein 1‐like kinase (CK1) 24, downstream checkpoint kinases 1 and 2 (Chk1/2) 25, 26 and homeodomain‐interacting protein kinase 2 (HIPK2) 27. Phosphorylation of the TAD has been shown to modulate its binding to MDM2 and p300, therefore acting on the regulation of p53 stability and transcriptional activity (for review 28). The DNA‐binding domain of p53 is also involved in mediating protein‐protein interaction. For example, three dimensional structures of the p53‐DBD in complex with two apoptotic factors, the apoptosis‐stimulating of p53 protein 2 (ASPP2) and the B‐cell lymphoma‐extra large (Bcl‐xL) have been solved and revealed that both factors interact with the DNA‐binding interface of p53 and therefore potentially compete with DNA‐binding in the nucleus (Fig. 1) 29, 30. Interestingly, ASPP2 and Bcl‐xL harbour opposite function in the regulation of p53‐mediated apoptosis, with pro versus anti‐apoptotic function, respectively. This suggests different mode of p53 regulation, such as transcription dependent/independent, despite involving similar binding properties. Communication between p53 domains has been shown to modulate p53 function. For example, binding of the N‐terminal proline‐rich region of p53 to its DNA‐binding domain increases p53 stability by inhibiting aggregation of the tetrameric form 31. Moreover, intermolecular tetramerization of p53 increases the strength of individual binding interactions trough avidity and is key for the regulation of p53 binding to the promoter region of its target genes and protein partners (for review 32). Indeed, p53 tetramerization markedly increases its ability to bind DNA compared to the monomeric form 33. Understanding the detailed molecular mechanisms of p53 interactions to its different binding partners is primordial in order to fully understand the key steps allowing p53 activation and subsequent regulation of cell fate determination.

**Figure 2:**
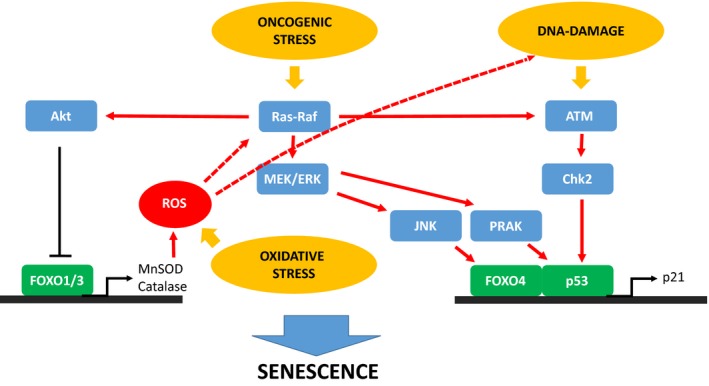
Schematic/pathway representation of the FOXO‐p53 interplay in cellular senescence. Ras‐mediated activation the PI3K/Akt signalling pathway leads to phosphorylation and inactivation of FOXO1/3 and therefore a decrease in expression level of the ROS detoxifying enzymes MnSOD and catalase. Increased ROS level affects two pathway (i) activation of the Ras/Raf signalling cascade (ii) DNA‐damage dependent activation of the ATM pathway. Upon oncogenic stress the Ras‐Raf pathway is also activated leading to MEK/ERK‐mediated activation of (i) JNK kinase which in turn phosphorylates and activates Foxo4 (ii) PRAK kinase which in turns phosphorylates and activates p53. p53 can also be activated *via* the ATM/Cdk2‐mediated DNA‐damage response. Activated p53 and FOXO4 can interact and upregulate the transcription of the senescence master regulator p21.

Because of its pro‐apoptotic function, p53 is recognized as tumour suppressor, and is found mutated in more than half of all human cancers affecting a wide variety of tissues 34. p53 acts as a sensor for cellular stresses such as genotoxic stress, hypoxia, telomere loss or oncogenic signalling. In absence of cellular stress signals, p53 can directly interact with MDM2 *via* its N‐terminal transactivation domain 35, 36. MDM2 is an E3 ubiquitin‐protein ligase which acts in concert with ubiquitin‐activating (E1) and ‐conjugating (E2) enzymes in order to poly‐ubiquitinate at least six reported lysines within the C‐terminal tail of p53 (Lys‐370, Lys‐372, Lys‐373, Lys‐381 and Lys‐386) 37. However, it is yet unclear how MDM2 binding at the N‐terminal part of p53 results in ubiquitination of its C‐terminal tail. Molecular details of MDM2 binding in context of the full‐length p53 in the future might help to fully understand the exact mechanisms of MDM2 regulation of p53. Poly‐ubiquitinated p53 is recognized by the 26S preoteasome and subjected to proteasomal degradation 38, 39, 40. Upon DNA‐damage induced by diverse stresses stimuli, p53 is post‐translationally modified leading to MDM2 displacement from p53 thereby stabilizing and activating p53. Activated p53 can activate the transcription of genes involved in specific anti‐proliferative response, including transient cell‐cycle arrest, apoptosis or cellular senescence (for review 18). The molecular mechanisms allowing p53‐dependent regulation of cellular senescence induced by intense oncogenic signals or replicative stress have been extensively studied in the last decades. Replicative senescence is induced by progressive telomere shortening that is recognized by the cell as double‐strand breaks and result in the activation of the DNA damage response 10, 11, 12. Overexpression of p53 in mice with deficient telomerase activity results in activation of senescence and reduced tumour progression whereas p53 inactivation leads to senescence bypass and tumour development 41, 42. Mechanistically, ATM activation at sites of DNA double strand breaks leads to direct phosphorylation of p53 at Serine 15 (Ser15). Moreover, ATM phosphorylates and activates Chk1/2. Upon activation Chk1/2 phosphorylate p53 at Ser20 23, 25, 26, 43, 44, 45, 46 which in turn protects p53 from MDM2‐mediated ubiquitination and subsequent proteasomal degradation. p53 phosphorylation at Ser15 or Ser20, however, does not alter its ability to interact with MDM2. This is further supported by the three dimensional structure of p53‐MDM2 complex showing no direct contacts between these two p53 serines and MDM2 35. Nevertheless, *in vitro* phosphorylation assay using recombinant kinase showed that Ser15 phosphorylation of p53 is required for CK1‐mediated phosphorylation of p53 at Thr18 which in turn strongly impairs MDM2 binding to p53 24. Upon stabilization, p53 can accumulate into the nucleus and activate the transcription of genes involved in the regulation of cellular senescence. Indeed, activated p53 transcriptionally upregulates p21 a cyclin‐dependent kinase inhibitor (Cdk) which in turn downregulates Cdk2‐mediated phosphorylated retinoblastoma protein (pRb) inactivation and cell‐cycle progression 43, 47, 48. In line with this, loss of p21 in normal human fibroblasts caused cell to bypass telomere‐dependent replicative senescence 49. In addition, p53 can induce cellular senescence in response to oncogenic signals. Expression of oncogenic mutated Ras (Ras V12) in primary fibroblasts results in p53‐dependent permanent cell cycle arrest 50. This was first explained as a result of activation of the DNA‐damage response. Indeed, ectopic expression of oncogenic Ras accelerates DNA replication of human diploid fibroblasts and subsequent accumulation of double‐strand breaks leading ultimately to ATM/p53/p21‐mediated activation of cellular senescence 14, 51. Consistently, knockdown of p53‐regulators, ATM and Chk2 or p53 itself prevents Ras‐induced senescence in human diploid foreskin fibroblasts 14. In contrast, other studies demonstrate that p53 also acts independently of the DNA‐damage response in order to activate the oncogene‐induced cellular senescence. Indeed, Ras also acts through p53 *via* regulation of the p38‐MAPK (mitogen‐activated protein kinase) pathway and activation of the p38‐regulated/activated protein kinase (PRAK) 52, 53. In turn, PRAK directly phosphorylates and activates p53 leading to cellular senescence 54. To summarize, p53‐mediated activation of cellular senescence can be triggered by diverse stress stimuli, either linked to replicative stress or oncogenic signals and leads, ultimately, to transcriptional activation of the senescence regulator p21 (Fig. 2).

## FOXOs and cellular senescence

### FOXO proteins

The forkhead box O family of proteins consists of 4 transcription factors that share a highly conserved DNA‐binding, the forkhead box domain (FH). The FH domain is surrounded by large N‐ and C‐terminal intrinsically disordered regions which are essential for the regulation of FOXO function (Fig. 1) 55. FOXOs control a plethora of cellular functions, such as cell growth, survival, metabolism and oxidative stress, by regulating the expression of several targeted genes 56. Three‐dimensional structures of FOXO1, 3 and 4 FH domains free and in complex with DNA have been reported and revealed a similar mode of DNA recognition among FOXOs 57, 58, 59. Regulation of FOXO transcriptional activity is mediated *via* direct interaction of its DNA binding domain with two consensus sequence, the Daf‐16 family member‐binding element (DBE) and the insulin‐responsive sequence (IRE) 60. Furthermore, genome‐wide ChIP‐seq profiling of FOXO3 and RNA polymerase II (RNAPII) occupancy provided evidences that FOXO3 acts as a transcriptional activator preferentially binding to the enhancer regions and recruiting RNAPII in order to in initiate gene transcription 61. Interestingly, *in vitro* experiments using nuclear magnetic resonance (NMR) spectroscopy showed that the C‐terminal disordered region in FOXO3 (residues 610–650), which is conserved in FOXO1 and 4 as well, can directly interact *in trans* with its DNA binding domain on a surface which overlaps with the DNA‐binding interface (Fig. 1) 62. Therefore the authors suggested that the transactivation domain of FOXOs can also back‐fold *in cis* on the Forkhead domain in the context of the full‐length protein. This raises the question whether FOXOs can adopt an auto‐inhibited conformation interfering with DNA‐binding, and whether co‐factors might regulate FOXO transcriptional activity through binding to these regions. The C‐terminal disordered region harbouring the auto‐inhibitory motif is considered as the FOXO transactivation domain because of its key role in interactions with transcriptional regulators, including p53 and others 62, 63, 64. Future studies on the molecular details of FOXO regulation by transcriptional regulators will help to understand their multi‐faceted biological function. Because of their anti‐proliferative and pro‐apoptotic functions, FOXOs have been considered as *bona fide* tumour suppressors. Concomitant with FOXOs function in the regulation of oxidative‐stress signalling, reactive oxidative species are largely involved in regulation of FOXO activity. ROS act on different levels of regulation in order to mediate FOXO‐mediated transcriptional activation of antioxidative enzymes (i.e., MnSOD and catalase). For example, ROS induce cysteine oxidation of both FOXO4 and the nuclear import factor Transportin‐1 resulting in formation of a covalent complex *via* disulfide‐bridges 65. In turn, upon ROS activation, Transportin‐1 is involved in the nuclear import of FOXO4 thereby promoting its transcriptional activity. The expression and activity of FOXOs are tightly controlled by post‐translational modifications such as phosphorylation, acetylation, methylation and ubiquitination. These modifications impact on FOXO stability, sub‐cellular localization and transcriptional activity. Several of these PTMs are involved in FOXO‐mediated regulation of cellular senescence including c‐jun N‐terminal kinase (JNK) and the Akt kinase. Akt is a downstream target of the phosphatidylinositol 4,5‐biphosphate 3‐kinase (PI3K) pathway. This pathway is activated in response to cytokines and growth factors 66. Akt‐mediated phosphorylation of FOXO 1,3 and 4 results in FOXOs binding to the nuclear export 14‐3‐3 protein and subsequent translocation of FOXOs from the nucleus to the cytoplasm and thereby inactivation of FOXOs transcriptional activity 67, 68, 69. In addition, Akt also induces the degradation of FOXOs *via* the proteasomal pathway 70, 71. Akt‐mediated inactivation of FOXO proteins is negatively regulated by the lipid phosphatase and tensin homolog (PTEN) which is involved in the degradation of PIP3, thus inhibiting Akt activation by the PI3K pathway 72. In contrast to PI3K/Akt inactivation of FOXOs, oxidative‐stress signalling can specifically activate FOXO4 *via* the JNK kinase. Oxidative stress results in activation of the small GTPase Ral 73. Activated Ral leads to phosphorylation and activation of the JNK kinase which in turn phosphorylates FOXO4 in its C‐terminal disordered extension at Thr447 and Thr451. JNK‐mediated phosphorylation of FOXO4 results in FOXO4 translocation in the nucleus and activates FOXO4 transcriptional activity 73, 74, 75. It is worth to note that JNK‐phosphorylation sites on the C‐terminal disordered part of FOXO4 are not conserved in FOXO1 and 3. To summarize, FOXO transcription factors are involved in the regulation of a plethora of cellular functions determining cell fate decision between survival and death and are therefore highly regulated by numerous enzymes and co‐transcriptional regulators. Still, detailed mechanistic studies are needed to fully understand the intricate networks of regulatory events determining FOXO activity. We will now focus on the specific function of FOXOs in the regulation of cellular senescence.

### FOXO4 and activation of senescence

Intense oncogenic signalling has been described as a potent tumour suppressive mechanism able to bypass tumour progression by triggering cellular senescence 14. Given FOXO's functions as tumour suppressor, several studies contributed to assess the role of FOXOs in oncogene‐induced senescence (OIS). In these studies, the B‐raf proto‐oncogene (BRAF) V600E variant has been used as model of OIS. Indeed, BRAF V600E variant is expressed in several tumours, especially in melanoma 76, 77. This mutation increase BRAF kinase activity resulting in increased mitogen‐activated protein kinase MEK‐associated signalling. Aberrant activation of BRAF oncogene leads to an increased cell proliferation and cell cycle progression but also ultimately to oncogene‐induced senescence 78. De Keizer *et al*., demonstrated an important role of FOXO4 in BRAF‐associated oncogenic signalling. Indeed, ectopic expression of FOXO4 in cancer cell lines expressing the BRAF mutant induces senescence 74. The oncogenic form of BRAF phosphorylates the JNK kinase *via* the MEK pathway. In turn, activated JNK can phosphorylate and activate FOXO4 leading to cell cycle arrest and induction of cellular senescence. This was associated with increased cellular ROS levels which also contribute to MEK‐JNK‐pathway associated active phosphorylation of FOXO4 73, thus acting as a positive feedback loop. Activation of FOXO4 is correlated with an increased transcriptional activation of p21 and subsequent activation of cellular senescence 74. Conversely, another study showed that in mice inactivation of FOXO4 results in senescence bypass and consequently restores oncogenic‐BRAF induced melanoma formation 79. FOXO4‐mediated activation of cellular senescence has also been observed in endothelial progenitor cells 80, 81. Ectopic expression of the cleaved high molecular weight kinogen (HKa) accelerates the onset of endothelial progenitor cells senescence and correlates with an increased level of intra‐cellular ROS. As discussed before, increased ROS levels result in JNK‐mediated activation of FOXO4 leading to cellular senescence. All these studies provide strong evidence that oncogenic signals can promote FOXO4 activation of cellular senescence which serves as a defence mechanism in order to prevent cell hyper‐proliferation. Therefore, apart from FOXO function in apoptosis, cellular senescence also contributes to make FOXO transcription factors potent tumour suppressors (Fig. 2).

### FOXO1, FOXO3 and resistance to senescence

Strikingly, opposite effect on senescence has been observed for FOXO1 and FOXO3. In cerebral microvascular endothelial cells (CMECs) replicative senescence is characterized by an increase in p27 levels and no significant change in p21 therefore promoting the cell cycle arrest in G1 phase 82. In senescent CMECs, activation of the PI3K/Akt signalling results in phosphorylation of FOXO3 which in turn re‐localizes from the nucleus to the cytoplasm and therefore inactivate FOXO3 transcriptional activity. Inactivation of FOXO3 in senescent CMECs is correlated with a decreased level of ROS‐detoxifying enzymes: the manganese superoxide dismutase (MnSOD) and catalase, resulting in increased cellular ROS level. Conversely, overexpression of FOXO3a suppressed the senescence process of CMECs under replicative stress by re‐activating the transcription of antioxidant and therefore inhibiting ROS generation 83. This primordial role of Akt as senescence inducer *via* inactivation of FOXO1 and 3 has been further confirmed in mouse embryonic fibroblast (MEFs) both for replicative senescence, oxidative stress induced senescence, and oncogenic Ras induced senescence 84. For all models of senescence, inactivation of Akt‐signalling and thereby activation of FOXO1 and 3 in MEFs confers resistance to senescence and is associated with an increased intracellular ROS levels. Consistently, inactivation of either FOXO1 or FOXO3 restores premature senescence of Akt‐deficient MEFs. Although, the specific role of FOXO4 in these models of senescence was not accessed, it is tempting to speculate that increased ROS levels due to FOXO1 and FOXO3 inactivation might be involved in specific JNK‐mediated activation of FOXO4 and induction of senescence as shown for the aforementioned study. ROS regulation of JNK‐mediated activation of FOXO4 and Akt‐mediated inactivation of FOXOs might lead to differential activation states of FOXO4 versus FOXO1 and 3, explaining the antagonistic role of FOXOs in senescence (Fig. 2). The molecular mechanisms linking JNK‐mediated phosphorylation of FOXO4 and activation of cellular senescence are unknown. Interestingly, JNK phosphorylation sites on FOXO4 are located next to its transactivation domain involved in the binding to several co‐regulators including p53 and others 62, 63. This raises the question whether JNK‐mediated phosphorylation of FOXO4 can influence the binding of these transcription regulators and therefore modulate FOXO4 transcriptional activity allowing regulation of cellular senescence. Antagonistic functions of FOXOs in cellular senescence depending on the cellular context and individual FOXO members could be a result of the intricate feedback mechanisms leading to differential FOXO activation. Indeed, continuous activation of growth factor signalling leads to activation of two signalling pathway, the PI3K and the oxidative‐stress pathway. As seen previously, both pathways harbour opposite effects on FOXOs, and are either triggering their inactivation or activation, respectively. Furthermore, FOXOs themselves are involved in the feedback‐regulation of these pathway. Therefore, the control of the balance between FOXO activation and inactivation is likely responsible for cell‐context specific differences in FOXO‐mediated regulation of cellular senescence.

## FOXO‐p53 axis and senescence

Overlapping functions of FOXOs and p53 in cell‐cycle regulation and tumour suppression are suggested by several studies in mice. Indeed, p53 knockout transgenic mice are prone to increased tumour progression including lymphoma and hemangiomas 85, 86. Concomitantly, FOXO1, 3 and 4 triple knockout mice are also characterized by thymic lymphoma and hemangiomas 87. These observations suggest that FOXOs and p53 play similar functions at least in suppressing tumorigenesis. Consistently, FOXOs and p53 share several downstream target genes including WIF1, FASLG, GADD45, PA26 and p21 88, 89. Interestingly, p53 and FOXOs recognize different sequence‐specific DNA‐binding element on the promoter site of their target genes suggesting that they can act in a cooperative manner in order to regulate gene transcription. Indeed, dual binding of p53 and FOXOs at common promoter sites could (a) increase affinity/specificity for DNA binding by modulating the structural conformation of DNA (b) modulate the binding of common transcriptional regulators such as p300/CBP and/or the transcriptional machinery. Given the importance of both FOXO4 and p53‐mediated transcriptional activation of p21 in induction of cellular senescence, one can suggest functional interconnection between the two proteins. Indeed, growing evidence illustrate several links between FOXOs and p53 signalling highlighting by the fact that FOXOs and p53 either share common post‐translational modifications or are involved in inter‐regulation of their specific PTMs and more importantly that they directly interact both *in vivo* and *in vitro*. Furthermore, p53 and FOXOs share similar biological functions notably in controlling the balance between cell death and survival therefore limiting cell proliferation in stressed cells. For these reasons, they are both considered as *bona fide* tumour suppressor.

### Co‐regulation of p53 and FOXOs by PTMs

Acting similarly as Akt, serum/glucocorticoid regulated kinase 1 (SGK1) is involved in inactivation of FOXOs. Indeed, SGK1 is also activated by the PI3K signalling pathway. PI3K‐mediated phosphorylation and activation of SGK1 promote cell survival. In turn, SGK1‐mediated phosphorylation of FOXOs induces FOXO translocation from the nucleus to the cytoplasm thereby suppressing FOXO‐dependent transcription and their function in cell cycle arrest and apoptosis 90. p53 is involved in the regulation of SGK1 at different levels. First, SGK1 is a downstream target gene of p53 and is upregulated upon p53 activation 91. Second, genotoxic stresses induce p53‐dependent activation of the extracellular signal‐regulated kinases 1 and 2 (ERK1/2) leading to SGK1 activation and subsequent inactivation of FOXO3 92. Third, SGK1 activates MDM2‐dependent degradation of p53 suggesting a fine‐tune feedback‐loop where p53 can control its own expression level as well as FOXO transcriptional activity 93. The E3 ubiquitin ligase MDM2 also harbours distinct functions involved in the regulation of both p53 and FOXOs. Indeed, despite its function in p53 poly‐ubiquitination and proteasomal‐mediated degradation, MDM2 also controls the expression level and activity of FOXOs. MDM2 can directly interact with FOXO4 and catalyses multiple events of FOXO4 mono‐ubiquitination. Expression of increasing amounts of MDM2 in human breast adenocarcinoma MCF‐7 cells results at a low amount of MDM2 in an increase in FOXO4 transcriptional activity whereas expression of high amounts of MDM2 show opposite effects 94. Furthermore, mono‐ubiquitination of FOXO4 is inducible by oxidative stress. Hydrogen‐peroxide treatment of hypothalamic A14 catecholamine cells induces mono‐ubiquitination of FOXO4 and results in FOXO4 re‐localization towards the nucleus and subsequent increase in transcriptional activity 95. In contrast to FOXO4, MDM2‐dependent ubiquitination of FOXO1 and 3 results in their proteasomal degradation and transcriptional inactivation in a p53‐dependent manner 38. The exact mechanisms involving p53 in this process remain to be discovered. The antagonistic function of MDM2 in FOXO4 versus FOXO1 and 3 activities is another clue that could help understanding the opposite functions of FOXOs in cellular senescence. It is also worth noting that the enzyme responsible for de‐ubiquitination of FOXOs and p53 is also identical and named USP7 95, 96 as well as the histone acetyltransferase p300 and the deacetylase sirtuin‐1 (SIRT1) which are involved in both FOXOs and p53 acetylation and deacetylation, respectively 97, 98, 99, 100, 101, 102.

### FOXO‐p53 interaction

Nemeto *et al*., provided the first evidence of FOXO binding to p53 while observing the effect of nutritional stress on SIRT1 expression in mammalian cells. SIRT1 is an NAD^+^‐dependent deacetylase involved in the de‐acetylation and regulation of the transcriptional activity of both FOXOs and p53 97, 98, 100. The authors observed that under starvation FOXO3 can activate the transcription of SIRT1 which is under the regulation of a p53‐dependent promoter therefore suggesting a physical interaction between FOXO3 and p53. They demonstrated that indeed p53 co‐immunoprecipitates with FOXO3 both *in vitro* and in human immortal HELA cells and is responsible for the starvation induced FOXO3/p53‐dependent activation of SIRT1 expression 99. This interaction was further characterized *in vitro* in order to map the binding site of p53 on FOXO3 and inversely. NMR spectroscopy and pull‐down experiments conducted on several deletion constructs of both p53 and FOXO3 demonstrate that both the C‐terminal transactivation domain and the Forkhead domain (FH) of FOXO3 contribute to the interaction with p53 and that the DNA binding domain (DBD) of p53 is sufficient for this interaction 62. As both DNA‐binding domains of FOXO3 and p53 are involved in the complex formation, this raises the question whether this interaction is compatible with FOXO3 and/or p53 binding to DNA and thereby to understand whether they can synergise or antagonize in order to activate the transcription of their targeted genes. Interestingly, the DNA‐binding domain of p53 is also involved in the interaction with MDM2. It would be interesting to test whether FOXOs binding to p53 interfere with MDM2 binding and therefore increase p53 stability. Furthermore, the Forkhead domain and C‐terminal disordered fragment of FOXO3 being conserved among the FOXOs, this raises the possibility that FOXO1 and 4 might also directly interact with p53. Our group, recently confirmed using NMR that indeed FOXO4 can also directly interact with p53 103.

### FOXO‐p53 and senescence

Given the importance of both FOXO4 and p53‐mediated transcriptional activation of p21 in induction of cellular senescence, it has been hypothesized that FOXOs and p53 could share similar molecular mechanism for the control of cellular fate and senescence. Indeed, several hypotheses could explain how p53 and FOXOs co‐regulate the transcription of common target genes: (a) direct interaction at the promoter site of p21 leading to enhanced transcriptional activity (b) co‐recruitment of common transcriptional co‐activators (c) inter‐regulation of their PTMs resulting in increased stability and nuclear localization. Molecular and structural studies on such networks are lacking in order to provide answer, nevertheless, several studies clearly demonstrate a join function of p53 and FOXOs in the activation of cellular senescence. In human endothelial cells, overexpression of constitutively active form of Akt results in cell growth arrest and senescence‐like phenotype reducing the lifespan of these cells and correlates with and elevation of intra‐cellular levels of p21. This mechanism is p53‐dependent as inactivation of p53 in this system impairs the transcriptional up‐regulation of p21 and subsequent senescence‐like cell growth arrest. Given the fact that FOXO proteins are direct targets of Akt signaling, the authors assessed the role of FOXO3 and demonstrated that constitutive activation of Akt inhibits the transcriptional activity of FOXO3 leading to a decrease in the expression of ROS detoxifying enzymes and therefore increase in intra‐cellular ROS level. The authors conclude that the increase in ROS levels promotes senescence‐like growth arrest *via* a p53‐mediated activation of p21 expression 104. Here again, the specific function of FOXO4 in this system was not assessed. Indeed, accumulation of ROS can lead to an activation of the DDR response and subsequent activation of p53 105, but could also lead to a specific activation of FOXO4 *via* the ROS/JNK and/or ROS/MDM2 pathways as discussed previously 73, 74, 75, 95 and possibly contributes to the observed senescence‐like phenotype. Recently Baar *et al*., characterized the specific function of FOXO4 in p53‐mediated senescence response. They first observed that ionizing‐radiation (IR) induced senescence of human IMR90 fibroblasts results in increase FOXO4 expression and no detectable change in FOXO1 and 3 compared to non‐senescent cells suggesting a specific function of FOXO4 in senescence. They showed that upon IR‐damage FOXO4 promotes senescence over apoptosis and maintains the viability of senescent cells by repressing their apoptotic response. Mechanistically, FOXO4 directly binds to activated p53 in promyelocytic leukemia (PML) bodies at the site of DNA‐damage 106 and activates the p53‐dependent transcription of the senescence‐associated p21 gene 103. It is worth noting that the promoter of the p21 gene is composed of a canonical FOXO target sequence flanked by two p53 binding sites. The exact molecular mechanisms allowing FOXO4‐dependent p53 activation of p21 transcription are unknown but one can speculate that dual binding of p53 and FOXO4 at the promoter site of p21 can stimulate its transcription compared to a situation where a single transcription factor is available. More studies have to be done to reflect if such mechanism is plausible and in turn how the formation of this potential ternary complex FOXO4/p53/DNA could stimulate gene transcription. FOXO4 and p53 transcriptional activity are tightly regulated *via* the histones acetyl‐transferase (HATs). Indeed, FOXOs can recruits CREB‐binding protein (CBP)/p300 to the promoter of its target genes leading to the transactivation of the targets and similarly for p53 101, 102, 107. FOXOs binding to p53 could stabilize the HATs complex formation and thereby FOXOs‐p53 dependent gene transcription. Alternatively, FOXO4 binding to p53 can interfere with p53 binding to different co‐repressors and therefore increase p53 activity. For example, FOXOs and MDM2 share a common binding site on p53. This raises the question whether FOXO4 can compete with MDM2 for p53 binding. Allosteric clashes between FOXO4 and MDM2 for p53 binding might lead to MDM2 displacement from p53 resulting in decrease p53 proteasomal degradation and increased stability.

## Targeting the FOXO‐p53 axis, therapeutic approach in age‐related diseases

### Link between cellular senescence, longevity and age‐related diseases

Aging is defined by a gradual dysfunction and progressive deterioration of multiple organ systems. Consequently, aging is the major risk factor for several diseases including neurodegenerative disorders 108, cardiovascular disease 109, diabetes 110 and cancer 6, 111. A causality between the accumulation of persistent senescent cells and the aging process has been made based on the observation that clearance of senescent cells can delay or prevent tissue dysfunction and extend healthspan in several mouse models. The first studies made used of progeroid BubR1 (BubR1^H/H^) mice in order to demonstrate the efficiency of senescent cells removal in counteracting the aging process. BubR1 mice harbour a huge variety of aging‐associated phenotypes including short lifespan that correlates with an accumulation of p16^Ink4a^ positive cells, a master regulator of senescence 112, 113, 114. In this model, either genetic loss of p16^Ink4a^ or selective removal of p16^Ink4a^ ‐expressing cells (*INK‐ATTAC*) contribute to attenuate the development of age‐related pathologies in tissues that accumulates these cells 115, 116. This role of senescence in aging was further confirmed in naturally aged mice model using the same *INK‐ATTAC* system. The authors could demonstrate that clearance of p16^Ink4a^ ‐senescent cells significantly prolongs the healthspan of these mice, delays age‐related deterioration of several organs including kidney and heart 117 and prevents bone loss 118. All these studies provide many clues that therapeutic approaches allowing to target and kill senescent cells could be an efficient way to extend lifespan. In this regard, several studies screened large library of existing chemical compounds in order to identify drugs able to counteract the senescence process in mice. These study allowed the identification of 5 compounds harbouring potent senolytic effect: (a) ABT‐263/ABT‐737/A‐1331852, specific inhibitors of the anti‐apoptotic BCL family of proteins 119, 120, 121 (b) dasatinib/quercetin, unspecific inhibitors of several kinases used in cancer therapy 122. These non‐targeted approaches suffer from the lack of specificity of these potential drug candidates and therefore could possibly be associated with several undesired side‐effects. Dasatinib and quercetin have been reported to be non‐specific toward senescent versus normal cells 119. Therefore, making use of the extended and growing knowledge of the senescence‐associated pathway in order to identify potential drug target seems to be a reasonable alternative approach.

### The FOXO‐p53 axis as a drug target

As previously discussed, Baar *et al*. 103, showed that FOXO4 can promote senescence by interacting with p53 at the sites of DNA‐damage thereby up‐regulating the transcription of the senescence master regulator p21. Therefore, they decided to design a peptide specifically targeting this interaction in order to prevent senescence. The specificity of this peptide arises from its design that is based on previously published data on the FOXO3/p53 interaction 62. In order to increase specificity, this peptide contains a region of the p53 binding site on FOXO4 which differs in primary amino‐acids sequence of FOXO1 and 3. The peptide has been designed in a D‐retro‐inverso (DRI) manner that has been shown previously to increase drug potency both *in vitro* and *in vivo* 123. We demonstrated using NMR that FOXO4‐DRI peptide competes with FOXO4 for p53 binding and therefore can efficiently interfere with the FOXO4 ‐ p53 interaction. In turn, when introduced to senescent human IMR90 fibroblasts the FOXO4‐DRI peptide reduces cell viability more than 10 times compared to non‐senescent IMR90 or other cell‐type. At low dose of administration FOXO4‐DRI appears to be more selective toward senescent cells than the pan‐BCL inhibitor ABT‐737 that highlight the efficiency of such targeted approach for drug design. Mechanistically, FOXO4‐DRI promotes nuclear exclusion of active p53. Once released in the cytosol active p53 induces cell‐intrinsic apoptosis by activating a caspase‐dependent pathway likely involved in previously reported transcription‐independent p53‐mediated apoptosis at the mitochondria 124. *In vivo* injection of FOXO4‐DRI in fast aged Xpd^TTD/TTD^ and naturally aged p16: MR mice decreases senescence and counteracts subsequent loss of renal function 103. To summarize, rational design of drug candidates targeting one specific protein‐protein interaction will allow to efficiently and electively target and selectively eliminate senescent cells. Moreover, it will provide insights into the mechanisms involved in FOXO4‐p53 co‐regulation of cellular senescence.

## Conclusions and perspectives

Given is role in aging and age‐related diseases, cellular senescence and associated signaling pathway have been extensively studied the past years. A better understanding of the exact molecular mechanisms involved in senescence is a key step toward the discovery of new drug that can efficiently and selectively target senescent cells in order to counteract age‐related pathology or more “utopically” in normal aging. In this way, we and co‐workers designed a peptide that interfere with FOXO‐p53 mediated senescence that showed great potency and selectivity in targeting apoptosis of senescent cells in mice. Based on this work, one can speculate on many other targets that could be used for similar therapeutic approaches. Indeed, interfering with (a) FOXO4 activation *via* the ROS/JNK or ROS/MDM2 pathway (b) nuclear translocation of active p53 (c) FOXO4 and/or p53 DNA binding to the promoter region of p21, could possibly lead to similar outcome. For this purpose, detailed structural information on binary complexes involved in the corresponding pathway are of great help in order to efficiently and selectively target one given interaction therefore limiting drug toxicity *in vivo*. Given the intricate networks in which p53 mediates the interaction with numerous binding partners, it remains to be studied how drug‐like molecules modulate binding of p53 to other p53 regulators. Therefore, atomic details of p53‐drug interactions are required in order to clarify the exact mechanisms leading to the clearance of senescent cells. Such structural and functional work will allow to optimize the binding properties, affinity/specificity of drug‐like molecules and to reduce potential off‐target effects.

## Data Accessibility

Research data pertaining to this article are located at figshare.com: https://dx.doi.org/10.6084/m9.figshare.6176126.
